# Miglitol improves postprandial endothelial dysfunction in patients with acute coronary syndrome and new-onset postprandial hyperglycemia

**DOI:** 10.1186/1475-2840-12-92

**Published:** 2013-06-19

**Authors:** Daisuke Kitano, Masaaki Chiku, Yuxin Li, Yasuo Okumura, Daisuke Fukamachi, Tadateru Takayama, Takafumi Hiro, Satoshi Saito, Atsushi Hirayama

**Affiliations:** 1 Division of Cardiology, Department of Medicine, Nihon University School of Medicine, 30-1 Ohyaguchi Kamicho, Itabashi-ku, Tokyo 173-8610, Japan; 2 Department of Cardiovascular Medicine, Keiai Hospital, Tokyo, Japan; 3 Department of Advanced Cardiovascular Imaging, Nihon University School of Medicine, Tokyo, Japan

**Keywords:** Postprandial hyperglycemia, Endothelial function, Reactive hyperemia peripheral arterial tonometry, Acute coronary syndrome, Alpha-glucosidase inhibitor, Miglitol

## Abstract

**Background:**

Hyperglycemia, a risk factor for development of cardiovascular disease, causes endothelial dysfunction. Alpha-glucosidase inhibitors (α-GIs) improve postprandial hyperglycemia (PPHG) and may have favorable effects on associated cardiovascular disease. Effects of α-GIs in patients with acute coronary syndrome (ACS) and PPHG remain unclear; thus, we assessed the effect of α-GI miglitol on endothelial function in such patients by digital reactive hyperemia peripheral arterial tonometry (RH-PAT).

**Methods:**

Fifty-four patients with ACS who underwent primary percutaneous coronary intervention were enrolled in the study: 36 with new-onset PPHG and 18 with normal glucose tolerance. Eighteen PPHG patients were given 50 mg of miglitol with each meal for 1 week. Endothelial function was assessed on the basis of the RH-PAT index (RHI) before and after the 1-week miglitol treatment. The other 18 PPHG patients and the 18 NGT patients were not given any anti-diabetic agent for 1 week, and endothelial function was assessed.

**Results:**

Postprandial RHI decreased significantly in patients with PPHG. Miglitol improved PPHG significantly; postprandial RHI also improved (*p* = 0.007). Significant inverse correlation was found between the postprandial change in RHI and postprandial fasting-to-60-minutes surge in glucose (r = -0.382, *p* = 0.009). Moreover, the improvement in endothelial function correlated with the reduced postprandial glucose surge achieved with miglitol (r = -0.462, *p* = 0.001).

**Conclusions:**

Postprandial changes in glucose are related to endothelial dysfunction in ACS. Miglitol-based improvement in PPHG appears to improve endothelial function. The effect of miglitol on glucose-dependent endothelial function might improve outcomes of ACS.

## Introduction

Several studies have surprisingly shown that intensive glycemic control with insulin or sulfonylurea does not reduce the mortality associated with cardiovascular events in persons with diabetes [1-3]. Furthermore, cardiovascular morbidity and overall mortality are associated with postprandial hyperglycemia (PPHG) rather than the fasting blood glucose level [4-6], and chronic hyperglycemia induces endothelial dysfunction [7-9]. In animal studies, repetitive PPHG has been shown to produce endothelial dysfunction and increase cardiac ischemia and reperfusion injury [10-12]. In addition, vascular endothelial dysfunction contributes to cardiovascular events and can be useful for identifying patients at high risk for ischemic heart disease [13-15]. Thus, PPHG may cause endothelial dysfunction and subsequent atherosclerosis, increasing the risk of cardiovascular events. However, it remains unclear whether PPHG actually worsens endothelial function of patients with acute coronary syndrome (ACS).

Alpha-glucosidase inhibitors (α-GIs) competitively and reversibly inhibit intestinal membrane-bound α-glucosidase required for degradation of disaccharides and complex carbohydrates in the upper part of the small intestine [16-18]. The effect of α-GIs on the intestinal membrane manifests as a reduction in PPHG. Thus, treatment with α-GI acarbose might have a favorable effect on endothelial function in type 2 diabetes patients with ischemic heart disease [19-22]. Miglitol is an α-GI with unique pharmacokinetic properties. It is absorbed rapidly and almost completely from the small intestine after oral administration. In an animal study, miglitol was shown to reduce myocardial infarct size [23]. Moreover, vascular function of patients improved when miglitol was administrated repeatedly [24]. Thus, compared to other α-GIs, miglitol can be expected to suppress PPHG more strongly and thereby reduce the incidence of cardiovascular events. However, little is actually known regarding the specific effects of miglitol on postprandial glycemia and endothelial function in patients with ACS.

We investigated endothelial function in patients with and without PPHG who had undergone primary percutaneous coronary intervention (PCI) for ACS. We then assessed the effects of miglitol on postprandial glycemia and endothelial function in the ACS patients with PPHG.

## Methods

### Study participants

We recruited 54 ACS patients, aged 20 to 79 years, who underwent successful primary PCI at Nihon University Itabashi Hospital, Tokyo, Japan, between April 1, 2009 and March 31, 2011. The patients were not previously diagnosed with type 1 (insulin-dependent) or type 2 diabetes mellitus (DM), not previously treated with insulin or oral anti-diabetic agents, not on diet therapy, and without a hemoglobin A1c (HbA1c) level > 7.9% (NGSP units) or fasting blood glucose > 200 mg/dL. Patients with severe myocardial infarction, heart failure, severe hepatic disease, renal insufficiency (serum creatinine > 2 mg/dL or treatment by hemodialysis), or collagen disease patients were excluded. The 54 enrolled patients were diagnosed as having (n = 36) or not having (n = 18) PPHG, i.e., normal glucose tolerance (NGT), according the postprandial glucose level at 60 minutes after loading of a test meal. PPHG was defined as a postprandial glucose level ≥ 130 mg/dL at 60 minutes. The test meal was that recommended by the Japan Diabetes Society working group [25] and consisted of 56.5 g of carbohydrate, 16.0 g of protein, and 18.0 g of fat for a total 460 kcal of energy (Kewpie, Tokyo, Japan). The test meal was served with a 100-mL glass of water and ingested within 15 minutes. Yoshino et al. reported that the peak glucose level is reached 60 minutes after test meal loading, and that the glucose level at 60 minutes after test meal loading correlated with that at 120 minutes after glucose loading. A blood glucose value of 140 mg/dL at 120 minutes after glucose loading corresponds to 130 mg/dL at 60 minutes after test meal loading [25-27]. Thus, PPHG was defined as a postprandial glucose level ≥ 130 mg /dL at 60 minutes after test meal loading. For all patients enrolled, previously prescribed medications and daily diet were maintained throughout the study period. The study was approved by the Ethics Committee of Nihon University School of Medicine, and all participants provided written informed consent.

### Study design

**Figure 1:**
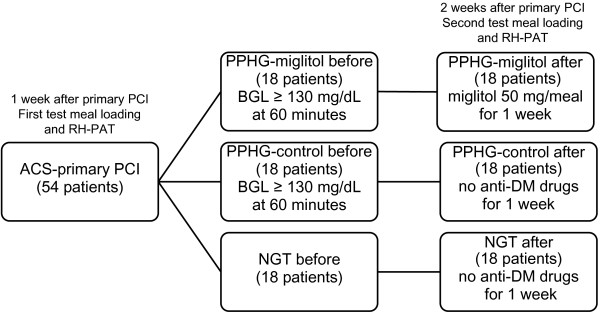
**Study flowchart.***ACS* acute coronary syndrome, *PCI* percutaneous coronary intervention, *BGL* blood glucose level, *PPHG* postprandial hyperglycemia, *NGT* normal glucose tolerance, *DM*: diabetes mellitus, *RH*-*PAT* reactive hyperemia peripheral arterial tonometry.

The study was a 1-week, randomized, single-blind controlled clinical trial. As shown in Figure 1, a baseline dietary tolerance test predicated on the test meal was performed 1 week after successful primary PCI to avoid any possible influence of ACS on dietary tolerance. All vasoactive medications were discontinued at least 12 hours prior to the test, and the test meal was given after a 12-hour overnight fast. We evaluated several biochemical markers associated with metabolism, inflammation, oxidative stress, and endothelial function in the fasting state and at 60 minutes and 120 minutes after test meal loading. The patients with PPHG were randomly divided into two groups; a PPHG-miglitol group (n = 18) that was given 50 mg of miglitol three times a day with each meal for 1 week and a PPHG-control group (n = 18) that was not given miglitol or any other anti-diabetic agent for 1 week. Upon completion of the 1-week miglitol intervention/non-intervention (control), the dietary tolerance test was repeated, and blood biochemical markers and endothelial function were re-assessed. The 18 remaining patients without PPHG (NGT group) also underwent a second dietary tolerance test at 1 week with re-assessment of blood biochemical markers and endothelial function.

### Laboratory tests

Standard blood samples were drawn in the fasting state to measure HbA1c, 1,5-anhydro-D-glucitol (1,5-AG), glycated albumin (GA), total cholesterol (T-CHOL), low-density lipoprotein-cholesterol (LDL-C), high-density lipoprotein-cholesterol (HDL-C), creatine phosphokinase (CPK), serum creatinine, estimated glomerular filtration rate (eGFR), and N-terminal prohormone of brain natriuretic peptide (NT-proBNP). Insulin resistance was evaluated by means of homeostasis model assessment of insulin resistance (HOMA-IR).

Plasma glucose, serum insulin, triglyceride (TG), high sensitivity C-reactive protein (hs-CRP), and derivatives of reactive oxidative metabolites (d-ROMs) as an oxidative stress marker measured with a free radical analytical system (FRAS4; H-&-D srl, Parma, Italy) [28] were measured in the fasting state and at 60 minutes and 120 minutes after test meal loading.

We also assessed incretins glucagon-like petide-1 (GLP-1) and glucose-dependent insulinotropic polypeptide (GIP) in the fasting state and at 60 minutes and 120 minutes after test meal loading. Blood samples were collected from 8 patients in the PPHG-miglitol group and 8 patients in the NGT group, and 10 μL of dipeptidyl peptidase-4 inhibitor per 1 mL blood was added immediately. The blood samples were centrifuged at 1,000 x g for 10 minutes under refrigeration. Total GLP-1 (9–36) was measured with an enzyme-linked immunosorbent assay (ELISA) kit (ALPCO Diagnostics Inc., Salem, NH, USA) and total GIP was measured with an ELISA kit (Millipore Corp., Billerica, MA, USA).

### Assessment of endothelial function

Endothelial function was evaluated in the fasting state and at 60 minutes and 120 minutes after test meal loading by digital reactive hyperemia peripheral arterial tonometry (RH-PAT) (Endo-PAT 2000, Itamar Medical Ltd., Caesarea, Israel). RH-PAT was performed as previously described [29,30]. Briefly, a blood pressure cuff was placed on the participant’s upper right arm; the left arm was used for control. A PAT probe was placed on the right and left forefingers. The RH-PAT protocol began with a 5-minute baseline measurement. The cuff on the test arm was then inflated to 60 mmHg above the baseline systolic pressure or to at least 200 mmHg for 5 minutes. The cuff was then deflated to induce RH. The RH-PAT data were digitally analyzed online (Endo-PAT 2000 software, ver. 3.0.4). The RH-PAT index (RHI), which reflects the extent of reactive hyperemia, was calculated as the ratio of the average amplitude of the PAT signal over 1 minute, starting 1.5 minutes after cuff deflation (occluded arm, A; control arm, C), divided by the average amplitude of the baseline PAT signal over a 2.5-minute period before cuff inflation (occluded arm, B; control arm, D). Thus, RHI = (A/B)/(C/D).

### Statistical analysis

Continuous variables are presented as mean ± SEM; disorders, e.g., hypertension, are presented as the number and percentage of patients affected; and use of various medications is presented as the number and percentage of patients receiving them. Between-group differences in data were subjected to one-way analysis of variance (ANOVA). Between-group differences in the prevalence of risk factors and use of medications were analyzed by chi-square test. Two-way ANOVA with repeated measures followed by Tukey’s post hoc test was used to detect significant changes in measured variables. Correlation was determined by linear regression analysis. All statistical analyses were performed with JMP, ver. 9 (SAS Institute, Cary, NC, USA). *p* < 0.05 was considered statistically significant.

## Results

### Baseline patient characteristics and blood test results

Baseline characteristics and laboratory values of patients are shown per study group in Table 1. There was no statistical between-group difference in age; sex; body mass index (BMI); prevalence of hypertension, hyperlipidemia, or chronic kidney disease; or history of smoking. There was no significant difference in the use of most medications (calcium channel blockers, beta blockers, angiotensin II receptor blockers [ARBs]/angiotensin converting enzyme inhibitors [ACE-Is], or statins). Neither was there any between-group difference in the hemoglobin, serum creatinine, eGFR, T-CHOL, HDL-C, LDL-C, TG, CPK, or NT-proBNP level. Use of nitrates, including nicorandil, was more prevalent in the NGT group than in the PPHG groups. The baseline HbA1c, GA, and HOMA-IR levels were significantly higher and the 1,5-AG level was significantly lower in the patients with PPHG than in the NGT patients. As expected, fasting plasma glucose levels were significantly higher in the patients with PPHG than in the NGT patients (Additional file 1: Table S1). However, no differences in fasting serum insulin, TG, and oxidative stress marker d-ROMs were noted between groups. In addition, the fasting RHI did not differ between groups (Additional file 1: Table S1).

**Table 1:** Baseline characteristics of study patients per group

	**PPHG-miglitol**	**PPHG**-**control**	**NGT**
	**n = 18**	**n = 18**	**n = 18**
**General characteristics**			
Age (years)	60.6 ± 2.1	65.1 ± 2.2	60.3 ± 2.1
Sex, male, n (%)	15 (83.3)	14 (77.8)	16 (88.9)
Body mass index (kg/m^2^)	25.1 ± 0.5	25.0 ± 0.7	25.3 ± 0.5
Hypertension, n (%)	15 (83.3)	16 (88.9)	16 (88.9)
Dyslipidemia, n (%)	15 (83.3)	14 (77.8)	14 (77.8)
Smoking, n (%)	12 (66.6)	11 (61.1)	10 (55.6)
Chronic kidney disease, n (%)	4 (22.2)	5 (27.8)	7 (38.9)
**Laboratory values**			
Hemoglobin (mg/mL)	13.6 ± 0.3	13.2 ± 0.2	13.1 ± 0.3
Creatinine (mg/mL)	0.82 ± 0.04	0.85 ± 0.04	1.01 ± 0.06
eGFR (mL/min/1.73m^2^)	69.5 ± 3.5	70.7 ± 2.6	63.1 ± 3.5
Hemoglobin A1c (%)	6.4 ± 0.1*	6.5 ± 0.1†	5.6 ± 0.1
1,5-AG (μg/mL)	11.4 ± 1.1**	11.5 ± 1.0††	20.8 ± 1.8
Glycated albumin (%)	15.4 ± 0.4*	15.2 ± 0.5†	14.2 ± 0.3
HOMA-IR	2.69 ± 0.38*	2.22 ± 0.47†	1.69 ± 0.17
Total cholesterol (mg/dL)	196.0 ± 12.2	195.5 ± 8.2	202.1 ± 7.1
HDL cholesterol (mg/dL)	45.8 ± 2.1	47.0 ± 2.1	46.0 ± 2.5
LDL cholesterol (mg/dL)	126.6 ± 12.0	125.3 ± 7.6	136.3 ± 5.7
Creatine phospho-kinase (max) (IU/L)	2360.6 ± 465.2	2453.3 ± 487.6	3174.7 ± 626.2
NT-proBNP (pg/mL)	666.3 ± 280.5	664.9 ± 238.6	616.7 ± 279.9
**Medications being used**			
Calcium channel blockers, n (%)	4 (22.2)	8 (44.4)	6 (33.3)
Beta blockers, n (%)	12 (66.7)	8 (44.4)	18 (44.4)
ACE-Is or ARBs, n (%)	13 (72.2)	12 (66.7)	12 (66.7)
Nitrates, n (%)	11 (61.1)**	12 (66.7)††	18 (100)
Statins, n (%)	15 (83.3)	14 (77.8)	14 (77.8)

Data are expressed as mean ± SEM or number and percentage of patients. *p < 0.05, PPHG-miglitol vs. NGT; **p < 0.01, PPHG-miglitol vs. NGT; †*p* < 0.05, PPHG-control vs. NGT; ††*p* < 0.01, PPHG-control vs. NGT.

PPHG-miglitol group: patients with postprandial hyperglycemia given miglitol for 1 week; PPHG-control group: patients with PPHG not given miglitol for 1 week; NGT group: patients with normal glucose tolerance; eGER: estimated glomerular filtration rate; 1,5-AG: 1,5-anhydro-D-glucitol; HOMA-IR: homeostasis model assessment of insulin resistance; HDL: high density lipoprotein; LDL: low density lipoprotein; NT-proBNP: N-terminal prohormone of brain natriuretic peptide; ACE-I: angiotensin converting enzyme inhibitor; ARB: angiotensin II receptor blocker.

There were no differences in baseline patient characteristics or biochemical markers between the PPHG-miglitol group and the PPHG-control group.

### Postprandial changes in blood glucose and insulin

**Figure 2:**
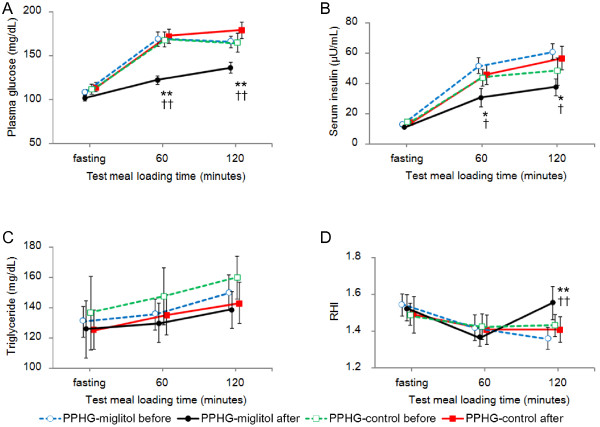
**Changes in plasma glucose levels (A), serum insulin levels (B), triglyceride levels (C), and RHI (D) in the PPHG groups, before and after the 1-week intervention (miglitol)/non-intervention (control).** Data are expressed as mean ± SEM. *p < 0.05, PPHG-miglitol before vs. PPHG-miglitol after; **p < 0.01, PPHG-miglitol before vs. PPHG-miglitol after; †*p* < 0.05, PPHG-miglitol after vs. PPHG-control after; ††*p* < 0.01, PPHG-miglitol after vs. PPHG-control after.RHI: RH-PAT index; PPHG: postprandial hyperglycemia; PPHG-miglitol before: patients with PPHG before miglitol administration; PPHG-miglitol after: patients with PPHG given 50 mg of miglitol every meal for 1 week; PPHG-control before: patients with PPHG before non-intervention; PPHG-control after; patients with PPHG after 1-week non-intervention.

Plasma glucose levels and serum insulin levels after overnight fasting and at 60 minutes and 120 minutes after the test meal are shown in Figure 2 and Additional file 2: Figure S1. With miglitol administration, postprandial plasma glucose levels were decreased significantly at 60 and 120 minutes after the test meal (from 175.2 ± 4.9 mg/dL to 132.2 ± 4.8 mg/dL at 60 minutes, *p* < 0.001; from 171.5 ± 6.6 mg/dL to 137.1 ± 6.1 mg/dL at 120 minutes, *p* < 0.001; Figure 2A), and postprandial serum insulin levels were also decreased significantly (from 47.1 ± 5.2 μU/mL to 27.0 ± 6.1 μU/mL at 60 minutes, *p* = 0.031; from 56.6 ± 5.8 μU/mL to 34.1 ± 5.5 μU/ml at 120 minutes, *p* = 0.015; Figure 2B). Plasma glucose levels and serum insulin levels improved significantly in the PPHG-miglitol group compared to those in the PPHG-control group. In the PPHG-control group and the NGT group, there were no significant time-specific changes in glucose or insulin levels between time point (Figure 2A and B, and Additional file 2: Figure S1A and B).

### Postprandial changes in lipids, oxidative stress and inflammatory markers

**Figure 3:**
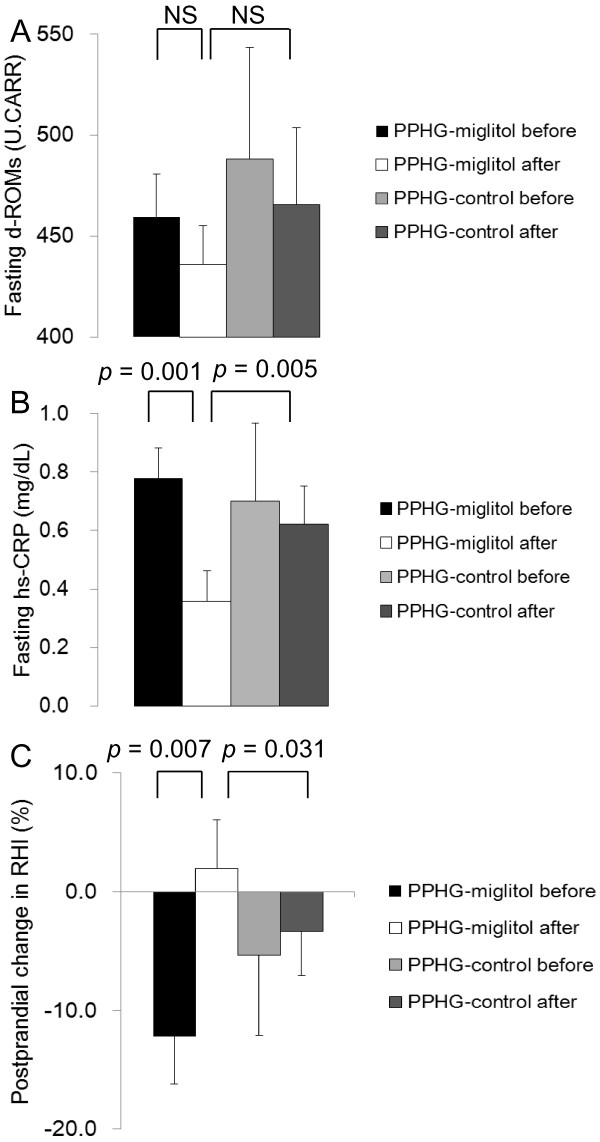
**Fasting d-ROMs levels (A), fasting hs-CRP levels (B), and postprandial percent changes in RHI (C) in the PPHG groups, before and after the 1-week intervention (miglitol)/non-intervention (control).** Data are expressed as mean ± SEM. d-ROMs: derivatives of reactive oxidative metabolites; hs-CRP: high sensitivity C-reactive protein; RHI: RH-PAT index; PPHG: postprandial hyperglycemia; PPHG-miglitol before: patients with PPHG before miglitol administration; PPHG-miglitol after: patients with PPHG given 50 mg of miglitol every meal for 1 week; PPHG-control before: patients with PPHG before non-intervention; PPHG-control after; patients with PPHG after 1-week non-intervention; postprandial percent change in RHI: (120 minutes RHI – fasting RHI)/fasting RHI × 100; NS: not significant.

The postprandial TG levels increased gradually in both the PPHG-miglitol group and the PPHG-control group with no between-group-difference (Figure 2C). There was no statistical difference in the postprandial TG or d-ROMs levels either before or after 1-week therapy between the PPHG-miglitol group and PPHG-control group (Figure 2C and 3A). However, in the NGT group, there was a significant time-specific decrease in the fasting d-ROMs level (from 469.8 ± 14.6 U.CARR to 367.0 ± 24.5 U.CARR, *p* = 0.004; Additional file 1: Table S1). Furthermore, the fasting hs-CRP level in the PPHG-miglitol group decreased significantly (from 0.778 ± 0.105 mg/dL to 0.359 ± 0.094 mg/dL, *p* = 0.001), and it also decreased significantly compared to that in the PPHG-control group (*p* = 0.005) (Figure 3B).

### Postprandial changes in endothelial function

Baseline fasting RHI in the NGT group was 1.62 ± 0.07, and neither RHI at 60 minutes (1.61 ± 0.08) nor RHI at 120 minutes (1.55 ± 0.05) differed from the fasting value (*p* = 0.834 and *p* = 0.285, respectively) (Additional file 2: Figure S1D). Nor was there a significant change in postprandial endothelial function after the experimental week. In other words, postprandial endothelial function was not significantly impaired in this group.

RHI did change significantly, however, in patients with PPHG (Figure 2D). In the PPHG-miglitol group, postprandial RHI decreased significantly from a baseline value of 1.56 ± 0.06 to 1.43 ± 0.07 by 60 minutes, and to 1.37 ± 0.06 by 120 minutes (*p* = 0.040 and *p* = 0.002, respectively). In the PPHG-control group, RHI also decreased significantly from a baseline value of 1.49 ± 0.13 to 1.41 ± 0.10 by 60 minutes, and to 1.41 ± 0.09 by 120 minutes (*p* = 0.049 and *p* = 0.048, respectively). RHI values at 60 minutes and 120 minutes were decreased significantly in patients with PPHG in comparison to the values in the NGT patients, suggesting significantly impaired postprandial endothelial function in both PPHG groups.

After miglitol administration, the fasting RHI was decreased at 60 minutes (from 1.53 ± 0.07 upon fasting to 1.37 ± 0.19 at 60 minutes, *p* = 0.019) but was restored to above the fasting level by 120 minutes (from 1.37 ± 0.07 to 1.56 ± 0.09, *p* = 0.031), and it improved significantly compared to that in the PPHG-control group (*p* = 0.041). We also evaluated postprandial endothelial function as percent change in RHI: [(120 minutes RHI – fasting RHI)/fasting RHI × 100]. The percent postprandial change in RHI was significantly suppressed after miglitol administration in the PPHG-miglitol group compared to that in the same group before miglitol administration and that in the PPHG-control group at 1 week (*p* = 0.007, *p* = 0.031, respectively; Figure 3C).

### Postprandial changes in incretins

We assessed incretins total GLP-1 and total GIP in the most recently enrolled patients in the PPHG-miglitol group (n = 8) and the NGT group (n = 8). After miglitol administration, total GLP-1 was significantly increased 60 minutes after the test meal (from 3.77 ± 0.43 pmol/L upon fasting to 4.58 ± 0.13 pmol/L at 60 minutes, *p* = 0.048; Additional file 3: Figure S2A). Moreover, the total GIP level was significantly decreased in patients in the PPHG-miglitol group 60 minutes after the test meal (from 514.7 ± 95.8 pg/mL upon fasting to 289.4 ± 58.6 pg/mL at 60 minutes, *p* = 0.006; Additional file 3: Figure S2B).

### Major determinant of postprandial endothelial function

No significant relation was found between fasting RHI and any other variable (age, sex, BMI, or level of serum creatinine, eGFR, HbA1c, 1,5-AG, GA, HOMA-IR, T-CHOL, HDL-C, LDL-C, CPK, NT-proBNP and fasting plasma glucose, serum insulin, TG, hs-CRP, or d-ROMs).

**Figure 4:**
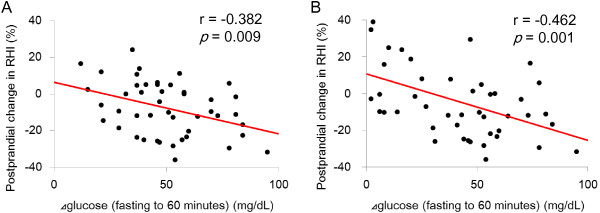
**Correlation between postprandial percent change in RHI and postprandial change in plasma glucose in the three groups before intervention (A), and in the PPHG**-**miglitol group before and after miglitol treatment (B).** Linear regression analysis revealed a significant inverse correlation between postprandial change in RHI and postprandial change in the glucose level (**A**). In the PPHG-miglitol group, there was a significant correlation between improvement in endothelial function and the reduction in glucose surge (**B**).

To clarify the determinants of postprandial endothelial dysfunction, we assessed relations between the postprandial change in RHI and other variables (age, sex, BMI, or level of serum creatinine, eGFR, HbA1c, 1,5-AG, GA, HOMA-IR, T-CHOL, HDL-C, LDL-C, CPK, NT-proBNP and postprandial changes in plasma glucose, serum insulin, TG, hs-CRP, and d-ROMs). The postprandial decrease in RHI correlated with the fasting-to-60-minutes surge in plasma glucose (r = -0.382, *p* = 0.009; Figure 4A), but no correlations was observed between the change in RHI and any other variable. In addition, we investigated correlation between the improved postprandial change in RHI and changes in other postprandial variables. Significant correlation was found between the postprandial improvement in RHI and the reduction in fasting-to-60-minutes glucose surge by miglitol administration (r = -0.462, *p* = 0.001; Figure 4B).

## Discussion

This is the first study to evaluate postprandial endothelial function on the basis of RHI in PPHG and NGT patients with early-phase ACS. It is also the first to investigate the effect of α-GI miglitol on RHI-determined postprandial endothelial function in ACS patients. Type 2 DM is a major risk factor for cardiovascular events [31-33]. However, several studies have shown that PPHG, more than impaired fasting glucose, is related to endothelial dysfunction and cardiovascular risk [4,34-39]. Impaired glucose tolerance is also known to cause ACS [40,41]. Vascular endothelial dysfunction may affect the initiation and progression of arteriosclerosis and cause coronary artery disease [42]. Postprandial hyperglycemic spikes have been suggested to induce endothelial dysfunction through an increase in hyperglycemia-induced oxidative stress. This may reduce production and bioavailability of nitric oxide (NO), since hyperglycemia-induced endothelial dysfunction is counterbalanced by arginine [43]. Previous studies have shown that oxygen-derived free radicals interfere with or destroy endothelium-dependent vasodilation by inactivating NO in normal vessels. In other words, a rapid glucose surge injures endothelial cells and suppresses NO production [44-47]. Results of the STOP-NIDDM trial, which showed that α-GIs reduce the incidence of cardiovascular events even in patients with impaired glucose tolerance, suggest the clinical importance of interfering with postprandial changes in glucose [19]. Thus, because repetitive PPHG is thought to cause cardiovascular events [46,48], ameliorating PPHG may affect endothelial function and prevent cardiovascular events [22,38,46]. In the present study, we observed a significant inverse correlation between postprandial endothelial function and PPHG. Thus, it is reasonable to think that amelioration of PPHG would play an important role in endothelial function. There were no postprandial changes in oxidative stress marker d-ROMs and no relation between the RHI and d-ROMs. However, d-ROMs remained higher in both PPHG groups than in the NGT group under the experimental conditions, suggesting that with repetitive PPHG, there is more exposure to oxidative stress. Hyperinsulinemia and insulin resistance have also been shown to cause endothelial dysfunction and to be risk factors for atherosclerosis [46,49-52]. However, the increased insulin after test meal loading may not have played an important role in the acute induction of endothelial dysfunction in our patients. We did not find a significant relation between the RHI and the serum insulin levels in our patients. Furthermore, our study did not show any relation between endothelial function and insulin resistance.

Evaluation of coronary endothelial function has made it possible to predict cardiovascular events. However, most of the evaluation methods used are invasive [53,54]. PAT can be used to noninvasively evaluate the endothelial function of resistance arteries and changes in the flow response resulting from endothelial-derived vasoactive substances including NO [55,56]. NO is an important factor contributing to augmentation of the PAT pulse amplitude after ischemia, and administration of endothelial NO inhibitor blunts the hyperemic response detected by PAT [56]. In addition, assessment by RH-PAT is independent of the examiner, and the probes are simply applied to the patient’s forefingers, one for control measurements. Moreover, the measured values are not affected by the autonomic nervous system. Previous studies have shown that measurement of endothelial dysfunction assessed by RH-PAT is related to cardiovascular risk [30] and that RH-PAT can be used to predict coronary endothelial dysfunction [15]. This makes it valuable for predicting coronary artery disease and cardiovascular events [15,57,58]. Because RH-PAT is a noninvasive, quantitative, and repeatable test, RH-PAT can serve as an important surrogate marker testing method for evaluating a patient’s vascular condition and the efficacy of treatment. The present study confirmed that RH-PAT measurements reflect coronary endothelial function after ACS. The fasting baseline RHI in our three patient groups was decreased significantly compared to the cutoff value (1.67), suggesting that endothelial function in patients with ACS is impaired. However, ACS did not influence postprandial endothelial function specifically, i.e., there were no time-specific changes under the experimental conditions. Moreover, we found a significant inverse correlation between postprandial endothelial function and PPHG, i.e., amelioration of postprandial glucose elevation suppressed postprandial endothelial dysfunction. Thus, measurement of endothelial function using RH-PAT may play an important role as surrogate marker.

Miglitol, in comparison to other α-GIs such as voglibose and acarbose, ameliorates the elevation in glucose shortly after test meal loading. This quick action may be associated with the unique pharmacokinetic properties of miglitol, i.e., rapid and almost complete absorption via the small intestine [17,18,59]. Therefore, miglitol may have a powerful inhibitory effect against α-glucosidase without additional adverse effects. It has been documented that α-GIs reduce cardiovascular events in patients with impaired glucose tolerance, suggesting the importance of interfering with postprandial glucose fluctuation [19]. Dramatic changes in endothelial function were observed with administration of miglitol. Not only was PPHG improved, but so was postprandial endothelial dysfunction. Our study showed that the glucose elevation between fasting and 60 minutes after test meal loading is significantly associated with postprandial endothelial function and that a reduction in the glucose surge indicates improved endothelial function. However, the effect of miglitol on endothelial function may not be totally dependent on the glucose level. In fact, although miglitol improved postprandial glucose levels measured at both 60 minutes and 120 minutes, endothelial function improved only between 60 minutes and 120 minutes. The below-baseline endothelial function at 60 minutes may be related to the glucose surge, but this decreased endothelial function was restored to the baseline fasting level by 120 minutes even though the blood glucose level remained high. Our data indicate that miglitol has a potent ameliorative effect on endothelial function in patients with ACS and PPHG, and the beneficial effects may not be totally dependent on postprandial glucose swings, but may rather be related to an additional pharmacologic mechanism of action. Incretins, especially GLP-1, secreted as a result of miglitol administration are thought to play an important role in endothelial function [60,61]. Oral administration of miglitol has been shown to increase GLP-1 secretion [62-64], and GLP-1 upregulates NO production in endothelial cells [65], independently improving vascular function [66]. Results of the present study suggest that increasing GLP-1 and decreasing GIP might be important to postprandial endothelial function and to the time course of improvement. Moreover, the present study showed that miglitol decreases hs-CRP, suggesting miglitol suppresses inflammation and may act synergistically in preserving endothelial function.

There were some limitations to the study. First, the lack of significance in the levels of various markers could have resulted from the relatively small patient groups. The results should therefore be interpreted with caution. Second, this was a very short-term follow-up study; the long-term effects of miglitol on endothelial function and ACS outcomes remain unknown. Third, in this study, we excluded patients with type 1 DM and type 2 DM who were treated with insulin and oral anti-DM agents and patients with high HbA1c. We speculate that even if such patients were included, we might have seen postprandial endothelial dysfunction related to postprandial glucose elevation. Fourth, we excluded patients with heart failure resulting from severe myocardial infarction, because it has been reported that diabetes can lead to heart failure after myocardial infarction, and moreover, endothelial function may be impaired in patients with heart failure [67-70]. Fifth, incretins, especially active GLP-1, are unstable in vivo; they dissolve immediately. Thus, it is difficult to investigate the clinical effects of incretins. Furthermore, we assessed incretins in only a few subjects, (8 PPHG-miglitol patients and 8 NGT patients), so the results are inconclusive.

## Conclusions

Noninvasive assessment of fingertip endothelial function may be clinically useful. In our patients with ACS, postprandial hyperglycemia rather than the fasting glucose level appeared to affect postprandial endothelial function. Miglitol is effective against postprandial hyperglycemia, hyperinsulinemia, inflammation, and endothelial dysfunction. Long-term follow-up studies are needed to confirm these findings.

## Competing interests

The authors have no conflicts of interest to disclose.

## Authors’ contributions

DK participated in the study design, collected the data, and drafted the manuscript. MC participated in study design and data collection. DF and TT collected the data. YL, YO, TH, SS, and AH reviewed and edited the manuscript. All authors read and approved the final manuscript.

## Supplementary Material

Additional file 1: Table S1 Plasma glucose, serum insulin, TG, hs-CRP, d-ROMs, and RHI per study group upon fasting and 60 minutes and 120 minutes after test meal loading, before and after intervention/non-intervention. Click here for file

Additional file 2: Figure S1 Changes in plasma glucose levels (A), serum insulin levels (B), triglyceride levels (C) and RHI (D) in the NGT group before and after 1-week non-intervention. Data are expressed as mean ± SEM. NGT: normal glucose tolerance; NGT before: patients with NGT before non-intervention; NGT after: patients with NGT after 1-week non-intervention; RHI: RH-PAT index. Click here for file

Additional file 3: Figure S2 Postprandial incretin levels in patients with PPHG treated with miglitol: total GLP-1 (A) and total GIP (B). Data are expressed mean ± SEM. We draw a blood sample from each of 8 patients in the PPHG-miglitol group and the NGT group. *p < 0.05, PPHG-miglitol before vs. PPHG-miglitol after. PPHG: postprandial hyperglycemia; NGT: normal glucose tolerance; GLP-1: glucagon-like petide-1; GIP: glucose-dependent insulinotropic polypeptide; PPHG-miglitol before: patients with PPHG before miglitol administration; PPHG-miglitol after: patients with PPHG given 50 mg of miflitol every meal for 1 week. Click here for file
