# Supplementary material for: Miglitol improves postprandial endothelial dysfunction in patients with acute coronary syndrome and new-onset postprandial hyperglycemia
Source: Cardiovasc Diabetol. 2013 Jun 19;12:92. doi: 10.1186/1475-2840-12-92 (PMC3691582; doi:10.1186/1475-2840-12-92)
Supplement: Additional file 1: Table S1 — Plasma glucose, serum insulin, TG, hs-CRP, d-ROMs, and RHI per study group upon fasting and 60 minutes and 120 minutes after test meal loading, before and after intervention/non-intervention. [file 1475-2840-12-92-S1.docx]

**Additional file 1: Table S1 Plasma glucose, serum insulin, TG, hs-CRP, d-ROMs, and RHI per study group upon fasting and 60 minutes and 120 minutes after test meal loading, before and after intervention/non-intervention.**

|  |  | **PPHG-miglitol before** | **PPHG-miglitol after** | **PPHG-control before** | **PPHG-control after** | **NGT before** | **NGT after** |
| --- | --- | --- | --- | --- | --- | --- | --- |
| **Plasma glucose**  **(mg/dL)** | fasting | 114.1 ± 3.0** | 111.3 ± 3.1 | 116.0 ± 10.8‡‡ | 112.5 ± 5.7 | 97.2 ± 1.3 | 99.6 ± 5.2 |
|  | 60 minutes | 175.2 ± 4.9** | 132.2 ± 4.8†† | 176.0 ± 15.8‡‡ | 169.9 ± 8.4 | 126.2 ± 4.5 | 128.6 ± 4.3 |
|  | 120 minutes | 171.5 ± 6.6** | 137.1 ± 6.1†† | 182.4 ± 15.1‡‡ | 164.9 ± 10.8 | 118.5 ± 2.9 | 120.9 ± 4.3 |
| **Serum insulin**  **(μU/mL)** | fasting | 9.3 ± 1.1 | 7.3 ± 0.9 | 7.7 ± 1.7 | 7.9 ± 1.5 | 7.0 ± 0.7 | 5.4 ± 0.8 |
|  | 60 minutes | 47.1 ± 5.2 | 27.0 ± 6.1† | 39.4 ± 9.6 | 38.9 ± 6.4 | 52.6 ± 7.6 | 43.7 ± 7.1 |
|  | 120 minutes | 56.6 ± 5.8** | 34.1 ± 5.5† | 50.0 ± 13.7‡‡ | 43.3 ± 9.1 | 32.2 ± 4.6 | 27.0 ± 3.2 |
| **Triglyceride**  **(mg/dL)** | fasting | 131.8 ± 10.1 | 127.0 ± 18.9 | 135.8 ± 24.3 | 125.1 ± 13.1 | 123.1 ± 6.8 | 113.9 ± 15.1 |
|  | 60 minutes | 136.7 ± 10.2 | 130.6 ± 13.0 | 146.5 ± 18.8 | 135.4 ± 13.3 | 125.8 ± 7.0 | 120.4 ± 13.5 |
|  | 120 minutes | 150.6 ± 11.7 | 140.0 ± 12.2 | 159.0 ± 14.1 | 143.3 ± 13.7 | 132.8 ± 7.4 | 131.8 ± 15.9 |
| **hs-CRP**  **(mg/dL)** | fasting | 0.778 ± 0.105 | 0.359 ± 0.094††, §§ | 0.622 ± 0.266 | 0.702 ± 0.137 | 0.703 ± 0.157 | 0.533 ± 0.386 |
|  | 60 minutes | 0.752 ± 0.100 | 0.358 ± 0.095††, §§ | 0.620 ± 0.242 | 0.693 ± 0.124 | 0.689 ± 0.144 | 0.509 ± 0.390 |
|  | 120 minutes | 0.728 ± 0.098 | 0.359 ± 0.096††, §§ | 0.587 ± 0.241 | 0.669 ± 0.122 | 0.663 ± 0.140 | 0.498 ± 0.363 |
| **d-ROMs**  **(U.CARR)** | fasting | 459.6 ± 21.0 | 435.8 ± 19.2 | 488.3 ± 55.2 | 465.5 ± 38.4 | 469.8 ± 14.6 | 367.0 ± 24.5$$ |
|  | 60 minutes | 443.4 ± 20.8 | 436.5 ± 18.4 | 490.8 ± 48.9 | 447.9 ± 35.6 | 457.8 ± 14.9 | 346.9 ± 33.5$$ |
|  | 120 minutes | 442.9 ± 20.8 | 438.9 ± 21.2 | 491.0 ± 44.5 | 455.9 ± 35.4 | 457.8 ± 15.0 | 358.3 ± 29.7$$ |
| **RHI** | fasting | 1.56 ± 0.06 | 1.53 ± 0.07 | 1.49 ± 0.13 | 1.49 ± 0.06 | 1.62 ± 0.07 | 1.57 ± 0.07 |
|  | 60 minutes | 1.43 ± 0.07* | 1.37 ± 0.05 | 1.41 ± 0.10‡ | 1.43 ± 0.06 | 1.61 ± 0.08 | 1.60 ± 0.17 |
|  | 120 minutes | 1.37 ± 0.06** | 1.56 ± 0.09††, § | 1.41 ± 0.09‡ | 1.44 ± 0.06 | 1.55 ± 0.05 | 1.61 ± 0.20 |

Data are expressed as mean ± SEM. **p* < 0.05, PPHG-miglitol before vs. NGT before; ***p* < 0.01, PPHG-miglitol before vs. NGT before; †*p* < 0.05, PPHG-miglitol after vs. PPHG-control after; ††*p* < 0.01, PPHG-miglitol after vs. PPHG-control after; $$*p* < 0.01, NGT before vs. NGT after; §*p* < 0.05, PPHG-miglitol after vs. PPHG-control after; §§*p* < 0.01, PPHG-miglitol after vs. PPHG-control after; ‡*p* < 0.05, PPHG-control before vs. NGT before, ‡‡*p* < 0.01, PPHG-control before vs. NGT before.

PPHG: postprandial hyperglycemia; NGT: normal glucose tolerance; PPHG-miglitol before: patients with postprandial hyperglycemia before miglitol administration; PPHG-miglitol after: patients with PPHG given 50 mg of miglitol every meal for 1 week; PPHG-control before: patients with postprandial hyperglycemia before non-intervention; PPHG-control after: patients with PPHG after 1-week non-intervention; NGT before: patients with NGT before non-intervention; NGT after: patients with NGT after 1-week non-intervention; hs-CRP: high sensitivity C-reactive protein; d-ROMs: derivatives of reactive oxidative metabolites; RHI: RH-PAT index.
