# Supplementary figures and images for: Miglitol improves postprandial endothelial dysfunction in patients with acute coronary syndrome and new-onset postprandial hyperglycemia
Source: Cardiovasc Diabetol. 2013 Jun 19;12:92. doi: 10.1186/1475-2840-12-92 (PMC3691582; doi:10.1186/1475-2840-12-92)

## Slide 1
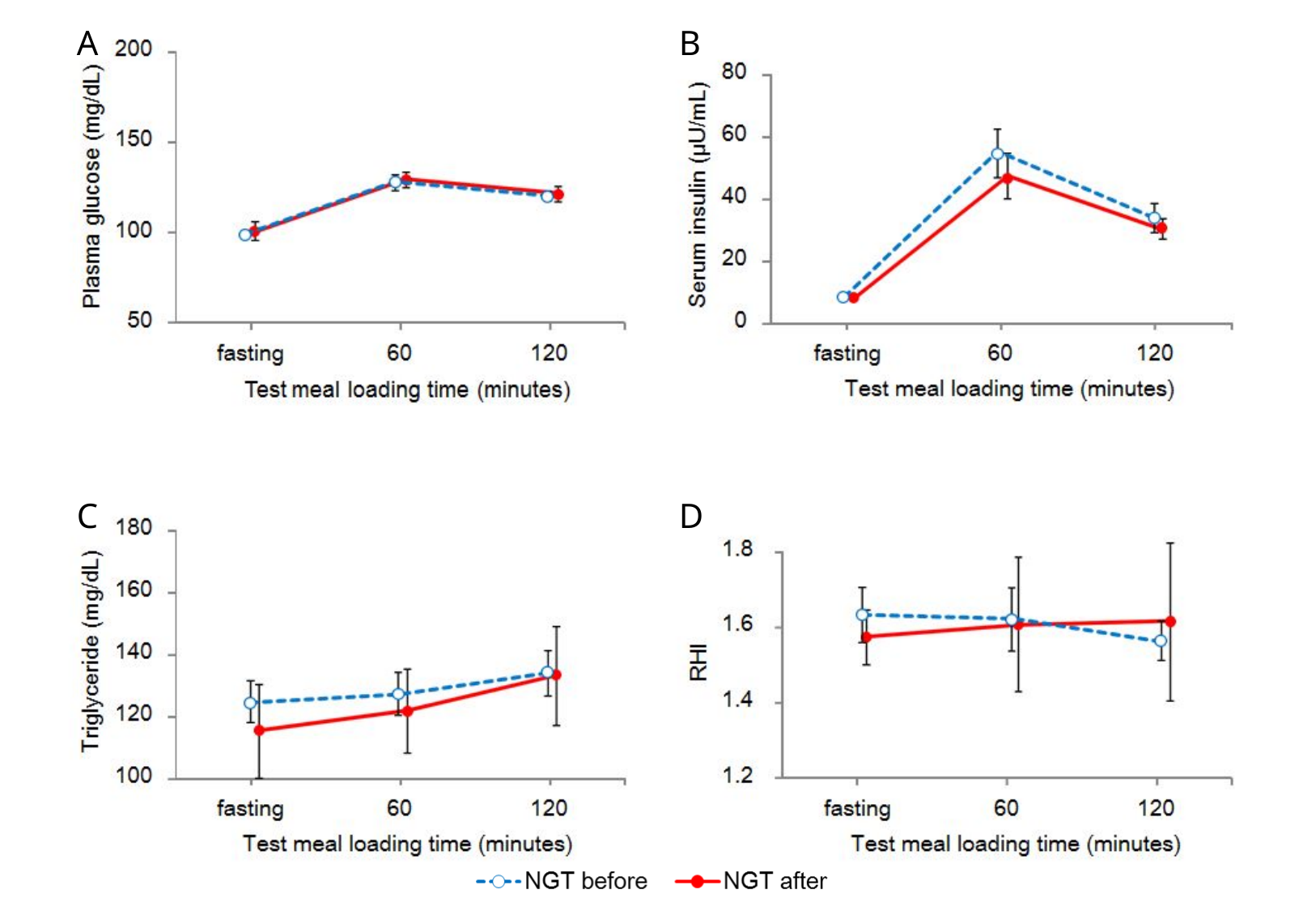

A
B
C
D

Supplement: Additional file 2: Figure S1 — Changes in plasma glucose levels (A), serum insulin levels (B), triglyceride levels (C) and RHI (D) in the NGT group before and after 1-week non-intervention. Data are expressed as mean ± SEM. NGT: normal glucose tolerance; NGT before: patients with NGT before non-intervention; NGT after: patients with NGT after 1-week non-intervention; RHI: RH-PAT index. [file 1475-2840-12-92-S2.pptx]

## Slide 1
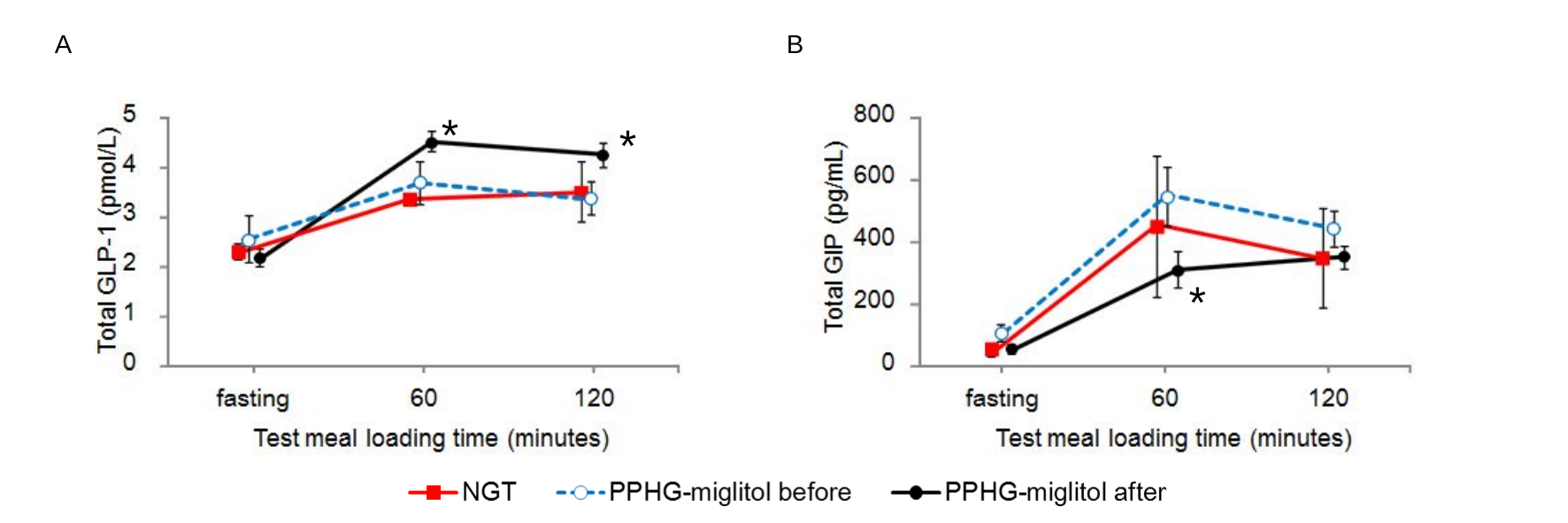

A
B
*
*
*

Supplement: Additional file 3: Figure S2 — Postprandial incretin levels in patients with PPHG treated with miglitol: total GLP-1 (A) and total GIP (B). Data are expressed mean ± SEM. We draw a blood sample from each of 8 patients in the PPHG-miglitol group and the NGT group. *p < 0.05, PPHG-miglitol before vs. PPHG-miglitol after. PPHG: postprandial hyperglycemia; NGT: normal glucose tolerance; GLP-1: glucagon-like petide-1; GIP: glucose-dependent insulinotropic polypeptide; PPHG-miglitol before: patients with PPHG before miglitol administration; PPHG-miglitol after: patients with PPHG given 50 mg of miflitol every meal for 1 week. [file 1475-2840-12-92-S3.pptx]
